# Supplementary material for: Causal Relationship between Aging and Anorexia Nervosa: A White-Matter-Microstructure-Mediated Mendelian Randomization Analysis
Source: Biomedicines. 2024 Aug 16;12(8):1874. doi: 10.3390/biomedicines12081874 (PMC11351342; doi:10.3390/biomedicines12081874)
Supplement: Supplementary file 1 [file biomedicines-12-01874-s001.zip › Supplementary Figure/Figure S7/Single SNP plot of MR analysis for ubm-b-1626 to AN.pdf]

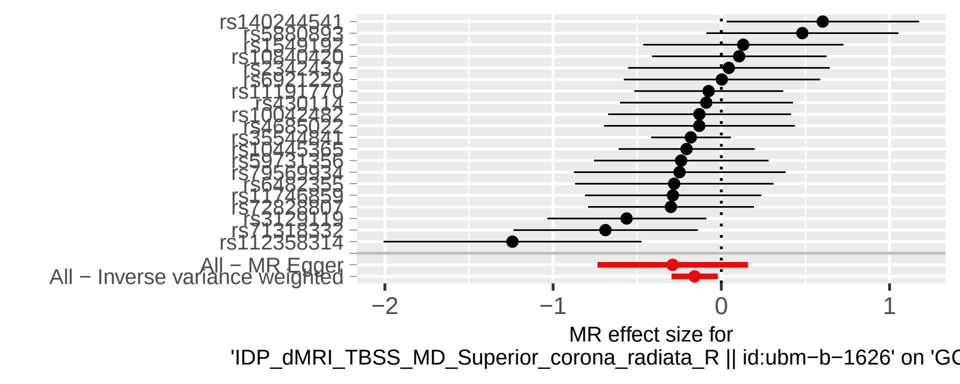

rs140244541
rs5880893
rs1549192
rs10840420
rs2342437
rs6921229
rs11191770
rs430114
rs10042482
rs4685022
rs35544841
rs10445365
rs59731356
rs79569934
rs6482355
rs11746859
rs72828807
rs3129119
rs71318332
rs112358314
All − MR Egger
All − Inverse variance weighted
−2
−1
0
1
MR effect size for
'IDP_dMRI_TBSS_MD_Superior_corona_radiata_R || id:ubm−b−1626' on 'G
